# Identification and functional analysis of glutamine transporter in *Streptococcus mutans*

**DOI:** 10.1080/20002297.2020.1797320

**Published:** 2020-08-04

**Authors:** Yuko Morikawa, Setsuyo Morimoto, Eri Yoshida, Shuhei Naka, Hiroaki Inaba, Michiyo Matsumoto-Nakano

**Affiliations:** Department of Pediatric Dentistry, Okayama University Graduate School of Medicine, Dentistry and Pharmaceutical Sciences, Okayama, Japan

**Keywords:** *Streptococcus mutans*, glutamine transporter, biofilm, membrane protein, *glnP*

## Abstract

**Background:**

*Streptococcus mutans*, a biofilm-forming bacterium, possesses several transporters that function as import/export molecules. Among them, the PII protein family is composed of members that regulate glutamine synthesis in bacterial species.

**Objective:**

In this study, we characterized the function of the glutamine transporter in *S. mutans* MT8148.

**Methods:**

The SMU.732 gene, corresponding to *glnP* in *S. mutans*, is homologous to the glutamine transporter gene in *Bacillus subtilis*. We constructed a *glnP*-inactivated mutant strain (GEMR) and a complement strain (comp-GEMR) and evaluated their biological functions.

**Results:**

Growth of GEMR was similar in the presence and absence of glutamine, whereas the growth rates of MT8148 and comp-GEMR were significantly lower in the presence of glutamine as compared to its absence. Furthermore, biofilms formed by MT8148 and comp-GEMR were significantly thicker than that formed by GEMR, while the GEMR strain showed a significantly lower survival rate in an acidic environment than the other strains. Addition of n-phenyl-2-naphthylamine, used to label of the membrane, led to increased fluorescence intensity of MT8148 and GEMR, albeit that was significantly lower in the latter.

**Conclusions:**

These results suggest that *glnP* is associated with glutamine transport in *S. mutans*, especially the import of glutamine involved in biofilm formation.

## Introduction

A previous genomic analysis of *Streptococcus mutans* predicted four putative glutamine uptake systems, one of which is known to be associated with *glnP* [1]. GlnP is an ATP-binding cassette (ABC) transporter and trans-membrane protein that utilizes the energy of adenosine triphosphate binding to transport various substrates across membranes [2]. The importance of ABC transporters for bacterial virulence has been demonstrated in ABC-type manganese [3–6], iron uptake [7–9], and ammonium export [10] transport systems. Furthermore, several effects on virulence have been observed and are thought to be indirect effects caused by altered bacterial fitness of mutants deficient in one of the ABC transporter components [11]. Bacterial transport systems enable bacteria to accumulate needed nutrients and extrude unwanted products, thus allowing them to survive stress, and also create conditions conducive to growth and development [11].

*S. mutans*, a Gram-positive facultative anaerobic bacterium, is considered to be a major etiologic agent of human dental caries and reported to form biofilm known as dental plaque on tooth surfaces [12]. This organism possesses several transport proteins in the cell membrane for export and import of molecules [13]. Glutamine metabolism is of central importance to bacterial physiology, because glutamine is an important resource required for biosynthesis of a variety of nitrogen-containing compounds as well as protein synthesis [14]. Since uptake and regulation of glutamine are important for bacterial fitness, glutamine transport is of interest for investigation of metabolic pathways and considered to link bacterial fitness with bacterial virulence [15]. Analysis of the complete genome of *S. mutans* strain UA159 in the Oralgen database (http://oralgen.org.) showed that the SMU.732 gene corresponds to glutamine transport in *S. mutans*, as it is homologous to the glutamine transporter gene in *Streptococcus pneumoniae*. In a previous genomic analysis, *S. pneumoniae* was found to have at least six glutamine transporters [16]. Like that in *Lactococcus lactis* and *Bacillus subtilis*, glutamine uptake in pneumococci is regulated, at least in part, by the nitrogen regulatory protein GlnR and glutamine synthetase (GS) protein GlnA [17–19]. *S. mutans* metabolizes carbohydrates to adhere to and form biofilm on tooth surfaces, thus allowing the pathogen to tolerate rapid and frequent environmental fluctuations [20]. Oral biofilms are subject to numerous environmental fluctuations, such as nutrient availability, aerobic-to-anaerobic transitions, and pH changes [21]. Therefore, it is essential to study glutamine transporters because they play a crucial role in the uptake of nutrients by *S. mutans* in biofilm.

The various roles of glutamine uptake transporters have not been studied in detail. In the present study, we evaluated one of those transporters to elucidate its function related to uptake of glutamine, and also analyzed its operon and regulatory genes.

## Materials and methods

### Bacterial strains and culture conditions

*S. mutans* strain MT8148 (serotype *c*) was used in the present study [10]. Bacterial organisms were grown at 37°C in brain-heart infusion (BHI) medium or Todd-Hewitt (TH) medium, as well as on Mitis Salivarius agar, each obtained from Becton Dickinson and Co. (Franklin Lakes, NJ, USA). When spectinomycin- or erythromycin-resistant *S. mutans* strains were cultured, spectinomycin (1 mg/ml) or erythromycin (10 μg/ml) was supplemented as necessary.

*Escherichia coli* DH5α (Nippon Gene, Tokyo, Japan), used as the host strain for transformation of plasmid DNA, was cultured in Luria-Bertani (LB; 1% tryptone, 0.5% yeast extract, 0.5% NaCl) medium while LB agar plate was prepared by adding 1.5% agar. Ampicillin sodium (100a μg/ml) was added to the medium for subcloning using a pGEM-T Easy Vector (Promega Co., Madison, WI, USA), while erythromycin (500 μg/ml) was added for use of pVA838 [22]. All antibiotics were obtained from Wako Pure Chemical Industries (Osaka, Japan).

### Construction of GlnP-deficient mutant

We generated a plasmid for construction of a GlnP-deficient mutant, as follows. First, the internal DNA fragment of *glnP* was amplified by PCR with AmpliTaq® (Life Technologies, Grand Island, NY, USA) using the GlnP2-F and GlnP2-R primer sets (Table 1). The amplified DNA fragment was purified using phenol chloroform, then precipitated with ethanol and ligated into a pGEM-T Easy Vector (Promega Co., Madison, WI, USA) to generate pYM1. Next, the ending sequences of the restriction enzymes in each primer (EcoR1-pkn-F and EcoR1-pknR, respectively; Table 1) were added to an *erm* cassette derived from pVA838. The resultant plasmid pYM2 was digested with *Eco*RI to become linear at a unique site, then blunted and ligated with an *erm* cassette to generate pYM2.

**Table 1.** Primers used in this study.

Names	Sequence (5’ to 3’)
GlnP2-F	ATG AAG AAC AAA TTT AAA GCT CTG ATG CTG
GlnP2-R	TTA TTT AAT CCT CTT TTC TAA ACG TTT TGC
EcoR1-pkn-F	AAG AAT TCG TAA TTA AGA AGG AGT GAT TAC
EcoR1-pkn-R	GCC GCA AGG AAT TCA TAG AAT TAT TTC CTC
OPE1-F	ATG GCG CTT TTA TTT TAA TGT CAC CAT TAC
OPE1-R	TTT GAT AAC CCA CAG CAC CCA CGT CTC AAA
OPE2-F	ATA ACC ATC TAC AAT GAC CTT GCC GCT AGT
OPE2-R	GGA CAA ATG GAA GCC AGC CGC AGT TTA GGG
OPE3-F	GGC CTT TTT AAT AAT ATC CAC ATC AAT TCC
OPE3-R	CTA AAA ATC CGC CTC CTA GTT GCC CCA AAA
16s-F	GAT GCT TCT GGG TTC CAA GCT
16s-R	TTA CGA ACG ACT TCA TTT CCG G

Transformation to *S. mutans* MT8148 was performed using a protocol reported by Lindler and Macrina [23]. Overnight cultures of *S. mutans* MT8148 were inoculated into TH medium supplemented with 10% heat-inactivated horse serum (Invitrogen, Carlsbad, CA, USA) and incubated for 2 h. Approximately, 200 μg of pYM2 plasmid was added to growing liquid cultures and incubation was continued for 2 h at 37°C, after which the cells were collected by centrifugation at 2400 x *g* for 10 min at 4°C, then plated on Mitis Salivarius agar containing EM (5 μg/ml), and incubated anaerobically at 37°C for 48 h. One positive transformant, a *glnP-*deficient mutant (GEMR) strain, was selected for confirmation. Appropriate introduction of pYM02 into strain ΔglnP was then confirmed using primer extension analysis. Following chromosomal DNA extraction of the transformants, primer extension analysis was used to determine the *glnP* transcription sites in MT8148 using the primers listed in Table 1 (GlnP2-F and GlnP2-R). Agarose gel electrophoresis of the PCR product showed an amplified band of approximately 1200 bp. To generate a complement (GEMR-comp) strain, each mutant was transformed with plasmid pDL278 [24] containing an intact copy of the respective deleted gene.

### Bacterial growth rates

Each *S. mutans* strain was grown overnight at 37°C, then inoculated into TH medium alone or TH medium containing 10 mM glutamine, with the cultures performed in duplicate. Growth curves were determined by measuring changes in optical density at 550 nm at 1 h intervals using a spectrophotometer (GE Healthcare, Fairfield, CT, USA).

### Confocal laser scanning microscopy observation of biofilms

Quantitative and structural analyses of biofilms were performed using confocal laser scanning microscopy (CLSM), according to the method described by Kuboniwa *et al*. [25]. MT8148, GEMR, and comp-GEMR were separately cultured in 10 ml of TH medium overnight at 37°C, then centrifuged at 2400 × *g* for 5 min at 4°C and washed with distilled water. Next, the bacterial cells were labelled with 5 μl of 10-mM hexidium iodide (Invitrogen) and incubated in the dark for 15 min at room temperature. Each cell suspension was adjusted to an optical density of 0.1 at 600 nm in chemically-defined medium supplemented with 0.5% sucrose (Van de Rijn and Kessler 1980). Saliva was obtained from two of the authors and diluted 1:4 with MilliQ water to produce 25% saliva, then 100 μl of each suspension was added to a Lab-Tek Chambered #1.0 Borosilicate Coverglass System with 8 chambers (Nunc, Rochester, NY, USA) that had been coated with filtered 25% human saliva to allow for biofilm formation. The chambers were incubated at 37°C with light shielding in an anaerobic chamber for 24 h, after which chemically-defined medium supplemented with 0.5% sucrose was removed and 100 μl of PBS added.

Imaging was performed using an LSM 510 confocal laser scanning microscope (Version 4.2, Carl Zeiss MicroImaging Co. Ltd., Jena, Germany) at a laser wavelength of 543 nm and biofilm images of each sample were acquired from three random positions. The obtained confocal images were analyzed using Image J for Macintosh (Version 10.2, National Institute of Health, Bethesda, MD, USA).

### Sonic disruption assay

Sonic disruption assays were performed as previously reported by Kuboniwa et al. [26], with some modifications. The tested strains were inoculated into 5-ml portions of TH medium in 6-well polystyrene microtiter plates and allowed to form biofilms. After 18 h of incubation, the resulting biofilms were sonicated for 1 or 2 min using a Handy ultrasonic disruptor (UR-20P; Tomy Seiko, Tokyo, Japan) at an output level of 7 (output power, 25 W; oscillating frequency, 28 kHz; tip diameter, 2.5 mm). Immediately after sonication, supernatants containing floating cells were removed by an aspirator and the remaining biofilms were gently washed with PBS.

Attached cells were removed using a cell scraper and suspended in PBS, then inoculated onto trypticase soy agar plates at 37°C for 48 h and counted. The rate of cells remaining after sonication was calculated as the percentage relative to number of total biofilm cells for each strain.

### Acid tolerance assay

Bacterial survival results for MT8148, GEMR, and GEMR-comp in acid tolerance assays were analyzed using the method described by Hanna et al. [27], with some modifications. The tested strains were cultured overnight in TH medium containing 0.3% yeast extract (THYE), then diluted 10-fold in fresh THYE (pH 7.5), and incubated at 37°C until reaching the mid-log phase. Cells were harvested by centrifugation at 2400 × *g* for 5 min at 4°C, and resuspended in THYE adjusted to pH 7.5 and 5.0 using HCl for unadapted and adapted conditions, respectively, and then incubated at 37°C for 2 h. An acid tolerance response was considered valid. In addition, adaptation to acid tolerance was determined by response of the cultures following exposure to killing pH for 3 h. The findings were quantitatively confirmed in triplicate by plating cells before and after incubation at a killing pH of 3.5 on THYE plates. The results are shown as percentage survival rate, which was calculated as follows: (number of cells following incubation at pH 3.5/number of cells before incubation at pH 3.5) × 100 (%).

### Fluorescence efflux measurement

Fluorescence measurements were performed using the method described by Ocaktan *et al*. [28], with some modifications. Fluorescence probes are considered suitable for uptake experiments because they are non-fluorescent in an aqueous environment, but become strongly fluorescent in nonpolar and hydrophobic environments [28]. N-Phenyl-2-naphtylamine (NPN) is a fluorescence polarization probe known to be sensitive to plasma membrane surfaces [29]. MT8148, GEMR, and GEMR-comp were grown to an optical density of 0.4 at 550 nm in TH medium and pelleted by centrifugation at 2400 × *g* for 10 min at 4°C. The cells were then washed with 10 mmol/ml NaCl and 50 mmol/ml NaPB (pH 7.0), and resuspended in the same buffer. Before fluorescence probe labelling, cultures were adjusted to an optical density of 0.2 at 600 nm, then 1 ml of the adjusted sample was transferred to 13 × 100-mm test tubes (IWAKI, Shizuoka, Japan).

The adjusted samples were labelled with NPN reacted at a final concentration of 5 or 10 μg/ml and incubated with light shielding for 30 min. Following incubation, labelled cultures were centrifuged at 2400 × *g* for 10 min at 4°C, and the resultant pellets were washed twice with 500 μl of 10 mM NaCl and 50 mM NaPB (pH 7.0). Thereafter, 100 μl samples were plated in 96-well plates (Nunc, Roskilde, Denmark), and absorbance was determined using a Twinkle LB970 fluorometer (Berthold Technologies GmbH & Co. KG, Bad Wildbad, Germany) at wavelengths of 355 and 460 nm.

### *PCR analysis of* glnP *operon and adjacent genes*

To characterize the *glnP* operon, RNA was extracted from cells grown to the mid-exponential phase, as described above, then the RNA samples were treated for 15 min at 37°C with RNase-free DNase (Promega). SuperScript III reverse transcriptase (Invitrogen) and random primers (Promega) were used to obtain complementary DNA (cDNA) from DNA-free RNA. PCR assays of DNA (as a positive control), and cDNA and MilliQ (as a negative control) were then performed, using specific primers that spanned SMU.730 and SMU.731 (OPE1-F and OPE1-R), SMU.731 and SMU.732 (OPE2-F and OPE2-R), and SMU.732 and SMU.733 (OPE3-F and OPE3-R) (Table 1). In addition, real-time (RT)-PCR was performed to examine the expressions of SMU.731 and SMU.732 (*glnP*) in the presence of 2 mM glutamine.

### Statistical analysis

All quantitative data are expressed as the mean ± SD of at least three independent experiments. Statistical analysis of variance (Fisher’s PLSD) was used to compare mean values, with *P* values < 0.05 considered to indicate statistically significant differences.

## Results

### *Biological properties of* glnP *deficient-mutants*

**Figure 1.**
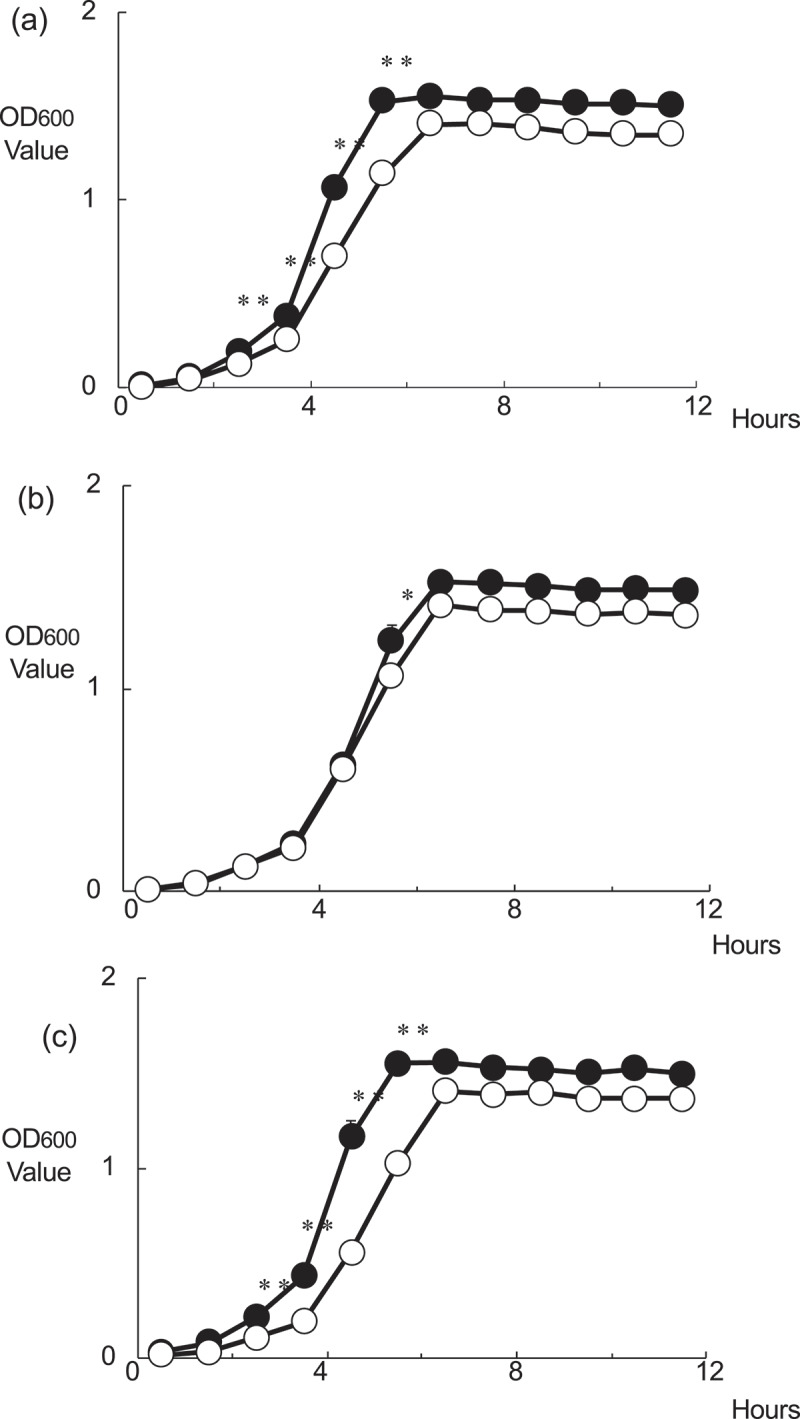
Bacterial growth rates of (a) *Streptococcus mutans* MT8148; (b) *glnP*-deficient mutant strain (GEMR), and (c) complement strain (comp-GEMR; mutant strain transformed with plasmid containing intact copy of deleted *glnP* gene) in Todd-Hewitt broth (THB) (●) or THB with 10 mM glutamine (○). Each data point represents the mean of three independent experiments. Growth rates differed significantly in cultures with and without 10 mM glutamine, as shown by Fisher’s protected least-significant difference analysis (*P < 0.01,**P < 0.001).

**Figure 2.**
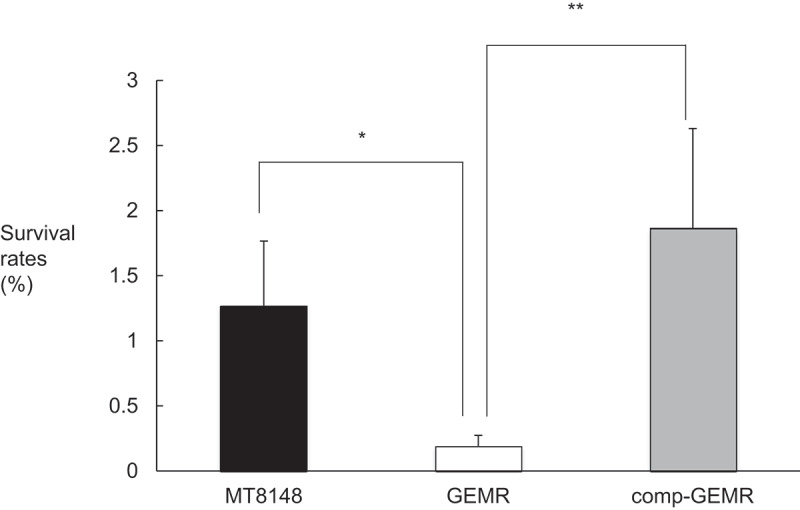
Acid tolerance assay results showing survival rates of *Streptococcus mutans* MT8148, *glnP*-deficient mutant strain (GEMR), and complement strain (comp-GEMR). Each data point represents the mean of three independent experiments. There were statistically significant differences between GEMR and the other strains, as shown by Fisher’s protected least-significant difference analysis (*P < 0.01,**P < 0.001).

There were significant differences in the bacterial growth curve rates for MT8148, GEMR, and comp-GEMR when cultured in THB alone (Figure 1(a)). When the GEMR strain was cultured in the presence of 10 mM glutamine, no change in growth rate was observed (Figure 1(b)), whereas growth of MT8148 and comp-GEMR was decreased significantly after 3 h in the presence of glutamine as compared with that in its absence (Figure 1(c)). These results indicated that the glutamine transporter in *S. mutans* is important for uptake of glutamine. Furthermore, acid tolerance assay results revealed that the GEMR strain was more sensitive to low pH, with the strain showing a significantly lower survival rate in an acid environment as compared to MT8148 or comp-GEMR (Figure 2).

### Biomass and structure of biofilms

**Figure 3.**
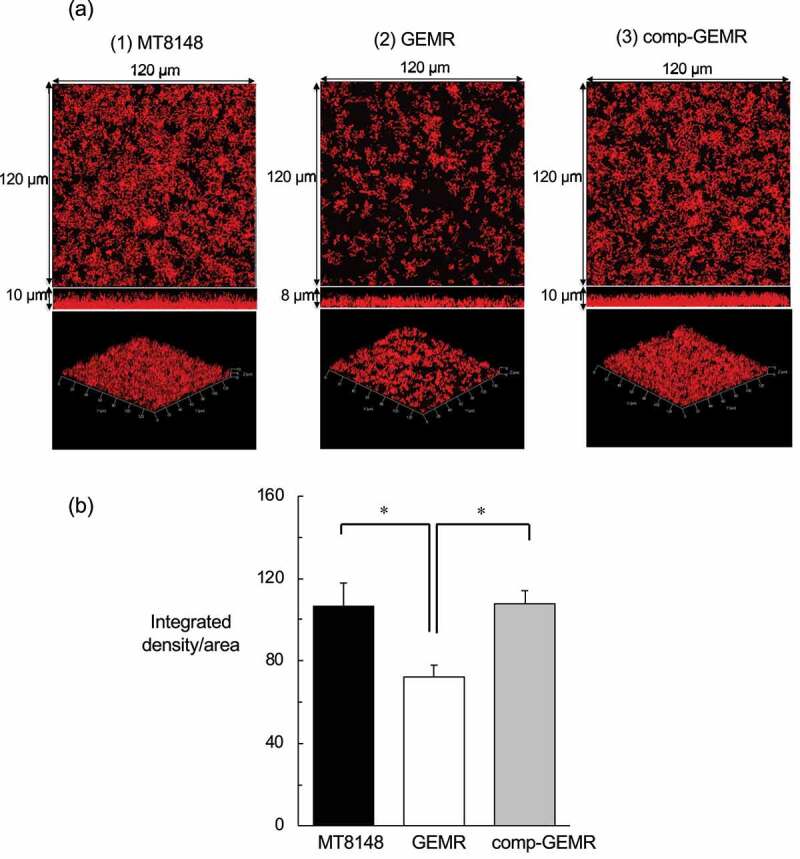
Analysis of biofilm formation. (a) Confocal laser scanning microscopic images of (1) *Streptococcus mutans* MT8148, (2) *glnP*-deficient mutant strain (GEMR), and (3) complement strain (comp-GEMR). (b) Densitometric analysis of biofilms generated by MT8148, GEMR, and comp-GEMR. Biofilm images of each sample were acquired using three random positions and three independent experiments were performed in triplicate with each strain. Statistically significant differences were noted among the strains, as shown by Fisher’s protected least-significant difference analysis (*P< 0.001).

**Figure 4.**
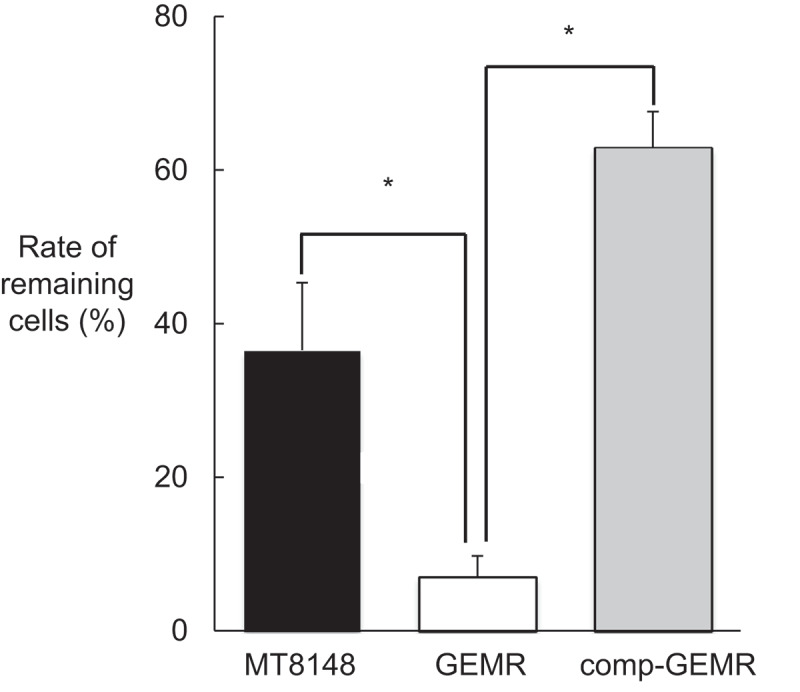
Tenacity of biofilms formed by *Streptococcus mutans* MT8148, *glnP*-deficient mutant strain (GEMR), and complement strain (comp-GEMR). The rate of remaining cells after sonic disruption was calculated as the percentage relative to number of total biofilm cells for each strain. Each data point shown represents the mean of three independent experiments. Statistically significant differences were noted between GEMR and the other strains, as shown by Fisher’s protected least-significant difference analysis (***P< 0.001).

We performed CLSM with hexidium iodide staining to examine *S. mutans* biofilms attached to the wells of polystyrene plates. The advantage of using a nucleic acid stain such as hexidium iodide in biofilm studies is that it provides sufficient intensity for visualization by confocal microscopy with minimum toxicity, thus maintaining cell viability [30]. According to our evaluation of CLSM images, biofilms formed by MT8148 and comp-GEMR were thicker than those formed by GEMR, while GEMR biofilms showed both small and large amorphous micro-colonies (Figure 3(a)). In addition, the biofilm mass formed by GEMR was less than that formed by MT8148 and comp-GEMR, which was supported by the results of quantitative assays (Figure 3(b)). Furthermore, to analyze the effect of import/export molecules on biofilm vulnerability, we compared the physical strength of the biofilms following brief ultrasonication (Figure 4), which showed that biofilms formed by GEMR were fragile as compared with those formed by the other strains.

### Analysis of exocytosis via GlnP

**Figure 5.**
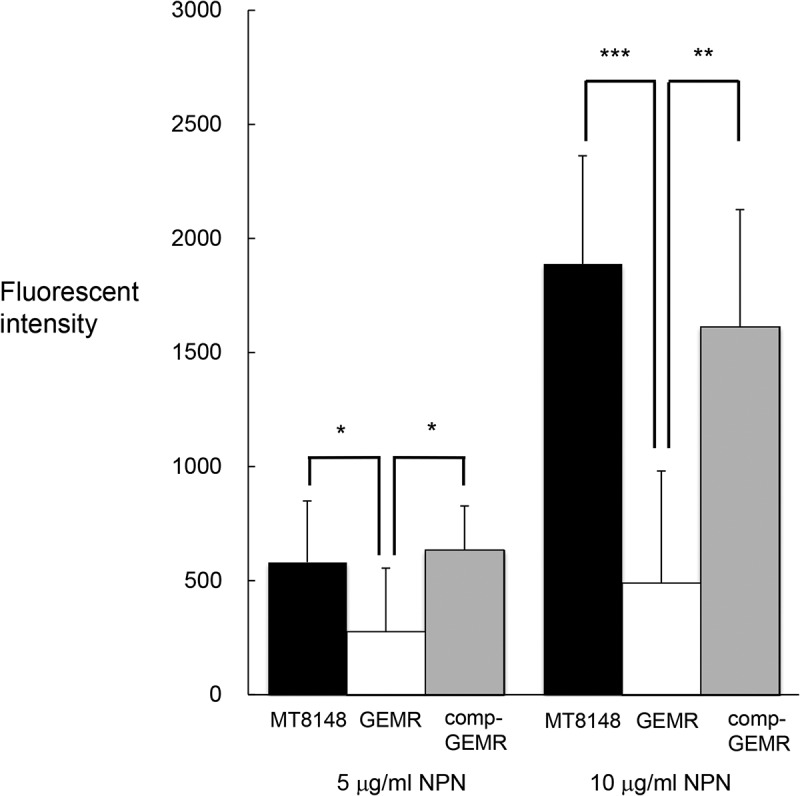
Analysis of exocytosis in *Streptococcus mutans* MT8148, *glnP*-deficient mutant strain (GEMR), and complement strain (comp-GEMR). Fluorescence of the cells was examined in the presence of different concentrations of the fluorescent probe *N*-phenyl-2-naphtylamine (NPN). Each data point shown represents the mean of three independent experiments. There were statistically significant differences among the strains as shown by Fisher’s protected least-significant difference analysis (*P< 0.05, **P< 0.01, ***P< 0.001).

Addition of 5 or 10 μg/ml of NPN led to an increase in fluorescence intensity of MT8148, GEMR, and comp-GEMR at 355 and 460 nm (Figure 5). However, the intensity of GEMR was significantly less than that of MT8148, suggesting a decrease to less than half of the amount the imported molecules. In addition, the wild-type phenotype was fully restored in comp-GEMR. Our analysis of exocytosis with NPN showed fewer molecules being released from the ammonium transporter in the plasma membranes. Based on the permease type and energy source, we speculated that the *glnP* gene has a function related to import of molecules, which may be one of the strategies used by *S. mutans* to respond to changes in its environment.

### *Predicting operon structure related to* glnP

Northern blot analysis of the transcriptional organization of the *glnP* gene locus detected a band specific to the *glnP* and SMU.731 genes, which was estimated to be approximately 2800 bp (data not shown), consistent with the 2800-bp band that spans the *glnP* and SMU.731 genes, as determined from the nucleotide sequence of the region.

**Figure 6.**
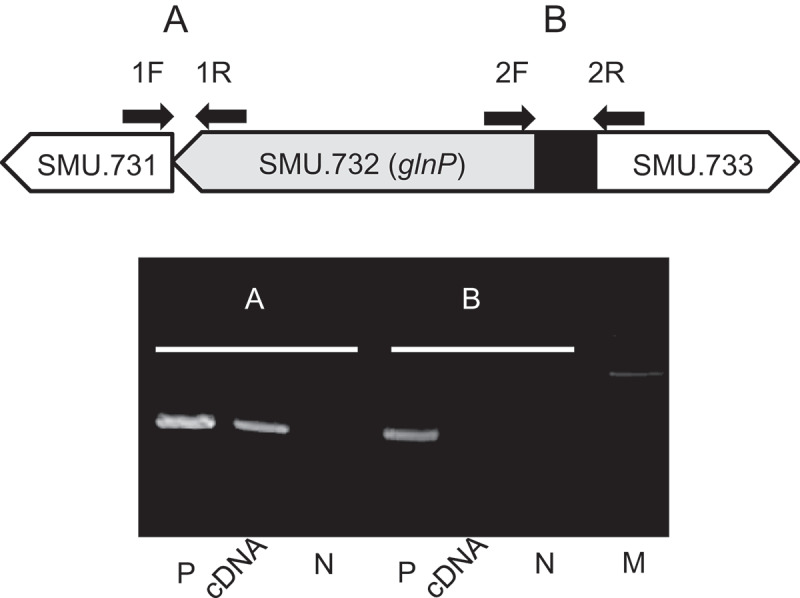
Evaluation of position and length of *glnP* and its operon in *Streptococcus mutans* MT8148. PCR analysis of the organization of the *glnP* operon and adjacent genes was performed using cDNA. The letters A and B correspond to the amplified regions illustrated above the electrophoresis gel. Lanes: M, 100 bp DNA ladder; P, chromosomal DNA of MT8148; cDNA, cDNA of MT8148, N, MilliQ water.

Transcriptional analysis using cDNA with specific primers showed that primer extension yielded an amplified band, indicating that *glnP* and SMU.731 constitute an operon. In contrast, no amplified bands were detected in the B region with cDNA, suggesting that SMU.733 is not part of the same operon as SMU.731 and *glnP* (Figure 6).

**Figure 7.**
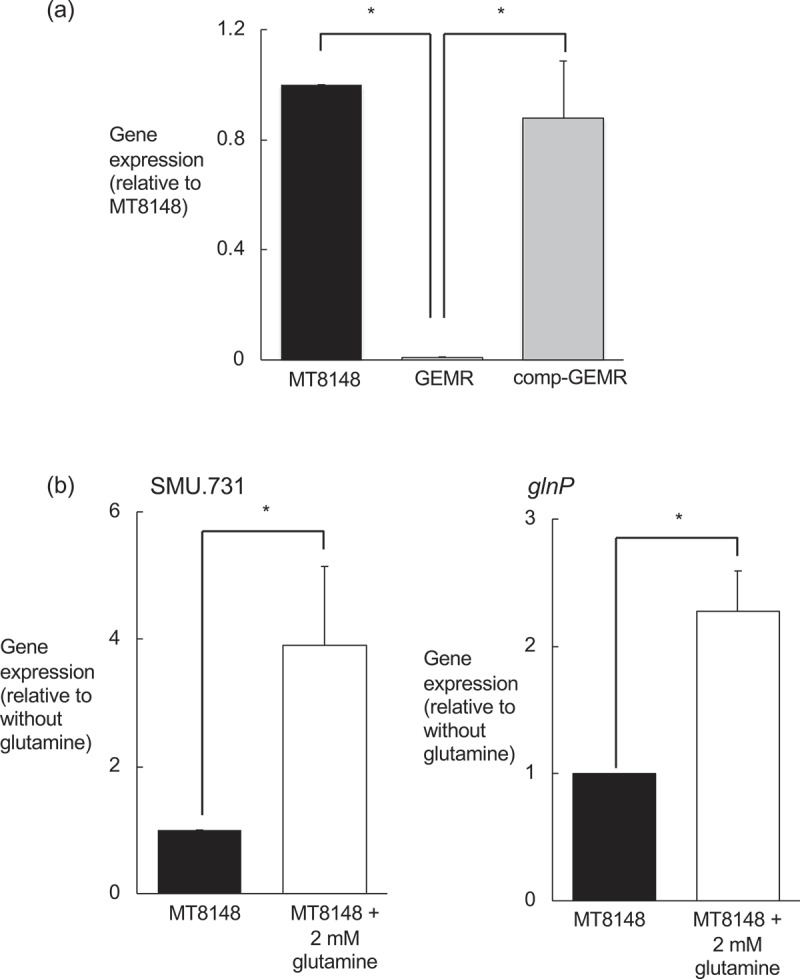
Evaluation of expressions of SMU.731 and *glnP*. (a) Expression levels of SMU.731 in *Streptococcus mutans* MT8148, *glnP*-deficient mutant strain (GEMR), and complement strain (comp-GEMR). Transcript levels were determined using real-time PCR with 16SrRNA as a control. There were statistically significant differences among the strains, as shown by Fisher’s protected least-significant difference analysis (***P< 0.001). (b) Real-time quantitative RT-PCR analysis of effect of glutamine on expressions of the SMU.731 and *glnP* genes. Three independent experiments were performed in triplicate with each strain. There were statistically significant differences for the expression of both genes detected between cultures with and without glutamine, as shown by Fisher’s protected least-significant difference analysis (*P< 0.05).

RT-PCR findings demonstrated that SMU.731 expression in GEMR was substantially decreased as compared with that in MT8148 (Figure 7(a)). In addition, the wild-type phenotype was completely restored in the complement strain. In the presence of 2 mM glutamine, expression of the *glnP* and SMU.731 genes was elevated relative to that in the absence of glutamine (Figure 7(b)). In *S. mutans* UA159, SMU.731 is located upstream of *glnP* and predicted to be a member of the PII protein family. That family is composed of proteins that regulate enzyme activity and gene expression, and are involved in nitrogen regulation as well as glutamine synthesis activities in bacterial species [31,32]. Our results suggested that the PII protein encoded by *glnP* regulates expression of the putative glutamine transporter.

## Discussion

*S. mutans* contains more than 280 genes associated with various transport systems, which are used by bacteria for survival and involved in uptake of ions, molecules, and carbohydrates [13]. In the present study, we performed functional analysis of the glutamine transporter in *S. mutans*. Using findings from a previous genomic analysis of *Streptococcus pneumoniae*, six putative glutamine uptake systems were predicted and shown by RT-PCR to be expressed under in vitro conditions [33]. In many bacteria, the requirement for glutamine uptake is fulfilled by glutamate dehydrogenase, which results in reductive amination of 2-oxoglutarate to glutamate [34]. *S. mutans* has been reported to utilize ammonia present in low levels in saliva to grow at the base of thick plaque deposits, where access to free amino acids and peptides might be limited [35]. We found that bacterial growth by the MT8148 and comp-GEMR strains was inhibited by glutamine, whereas GEMR showed no change in growth under the same conditions (Figure 1), suggesting that *glnP* encodes a protein that can import molecules. The increase or decrease in fluorescent intensity of NPN is sensitive to the state of membrane energization of the cell [29]. In the present study, the findings of decreased fluorescent intensity of NPN showed that inactivation of *glnP* blocked the import functions of *S. mutans*. Furthermore, our growth rate results demonstrated that GlnP is required for transport and utilization of glutamine by the bacterium.

Pneumococcal organisms produce large numbers of transporters that are involved in uptake and metabolism of sugars and amino acids, including classical phosphotransferase systems, ABC transporters, and ion gradient-driven transporters [16,36]. Glutamine metabolism is of central importance in bacterial physiology, because glutamine is an important resource for bacteria, as it is required for biosynthesis of a variety of nitrogen-containing compounds and protein synthesis. Thus, regulation of glutamine uptake and catabolism requires both general and specific regulators. Moreover, glutamine uptake and regulation are important for bacterial virulence [15]. Interestingly, a previous analysis of the *S. pneumoniae* genome predicted at least six putative glutamine ABC transporters distributed over the chromosome [16]. Like *Lactococcus lactis* and *Bacillus subtilis*, glutamine uptake in pneumococci is regulated, at least in part, by the nitrogen regulatory protein GlnR and GS protein GlnA [17,19,37]. In addition to GlnR and GlnA, pneumococcal organisms encode CodY, an ortholog of *B. subtilis* [2,38]. In *B. subtilis*, CodY, a member of the MerR family of DNA-binding regulatory proteins, functions as a global transcriptional regulator, and many CodY-regulated genes are involved in nitrogen and carbon metabolism [39,40]. Glutamate is synthesized from glutamate and ammonium, as part of an important process used by cells to assimilate nitrogen required for biosynthesis of all amino acids, which affects protein synthesis, as well as the structural and functional integrity of cells [41].

The ABC transporter in *S. mutans* is upregulated under nutrient deprivation to transport glutamate [42]. In addition, uptake of glutamate and its effects on two virulence attributes of *S. mutans*, acid production and acid tolerance, are important factors that affect the survival of the bacterium *S. mutans* and its persistence in plaque biofilm [42]. Because acid tolerance is an important virulence property of *S. mutans*, glutamate uptake might be linked to acid tolerance [41]. GEMR was shown to be more sensitive to acid than MT8148 and comp-GEMR (Figure 2). The present results also suggest that *glnP* is strongly related to acid tolerance. Therefore, biofilm formation and its structure may be directly affected by the function of the ABC transporter.

The nitrogen regulatory proteins PII and ABC transporter are found in most bacteria and often paired [43]. PII proteins regulate the activities of other proteins by protein–protein interactions, as shown by their role in modulating the activity of the transcriptional activator [44]. In *S. mutans* UA159, SMU.731 is located upstream of *glnP* and predicted to be a member of the PII protein family (Figure 6). In the present study, we obtained two lines of evidence suggesting that *glnP* and SMU.731 are co-transcribed as a single operon. Northern blotting was performed to characterize the *glnP* operon, which identified a band estimated to be approximately 2800bp, consistent with the length of *glnP* and SMU.731. In addition, a band amplified by cDNA was detected by PCR using primers spanning *glnP* and SMU.731, whereas no bands were detected with two primers spanning the up- and downstream locations of *glnP* and SMU.731. These results indicate that *glnP* and SMU.731 are co-transcribed as a single operon. Conservation of a genetic linkage in prokaryotes is a characteristic of operons that encode either essential cellular components or proteins that physically interact [45]. The PII protein plays a central role in signal transduction of the nitrogen-regulatory system in prokaryotes and controls transcription under nitrogen limitation conditions [46,47], while it has also been reported that bacterial ammonium transporter activity is regulated by nitrogen limitation [48]. Indeed, the location of the PII protein may play a crucial role in regulation of the expression or activities of glutamine synthase and nitrogenase [22].

Thus, we considered that the SMU.731 portion of the *glnP* operon may function as a regulatory gene. Furthermore, the present real-time RT-PCR assay results suggested that interaction between these genes is dependent on the presence of functional GlnP, because deletion of the *glnP* gene significantly reduced SMU.731 expression. SMU.731 may function as a receptor and transfer the signal to the *glnP* gene, suggesting a direct physical interaction between these proteins.

In summary, the present findings suggest that the *glnP* gene of *S. mutans* is essential for biofilm formation and export of molecules by the bacterium, and that SMU.731 may regulate expression of that gene. In addition, biofilm formation was shown to occur in response to the availability of nutrients supplied by the glutamine transporter. Additional studies are required to provide a better understanding of the role of the glutamine transporter in *S. mutans* in relation to the virulence of this pathogen.
